# Should schizoaffective disorder be diagnosed cross-sectionally (ICD-11) instead of longitudinally (DSM-5)? PRO

**DOI:** 10.1192/j.eurpsy.2021.37

**Published:** 2021-08-13

**Authors:** P. Falkai

**Affiliations:** Department of Psychiatry and Psychotherapy, Univeristy of Munich, Munich, Germany

## Abstract

Several changes to the classification of schizophrenia and other psychotic disorders have been made to increase the reliability, clinical use and validity of the diagnostic classification which are considered here. A diagnosis of schizoaffective disorder would only be made in ICD-11 when the definitional requirement of schizophrenia is met concurrently with mood symptoms that meet the definitional requirements of a moderate or severe depressive episode, a manic episode, or a mixed episode. This requirement is more restrictive compared to ICD-10, which just required the presence of symptoms of schizophrenia and mood disorder. The total duration requirement would be 4 weeks. A cross-sectional approach was maintained in the ICD-11 for schizoaffective disorders as there is no evidence on how a longitudinal “lifetime” criterion impacts cross-sectional inter-rater reliability, and the reliability of lifetime symptoms’ report by patients and retrospective assessment by clinicians remains unknown.

**Disclosure:**

No significant relationships.

